# Opportunistic consumption of coral spawn by the ruby brittle star (*Ophioderma rubicundum*)

**DOI:** 10.1002/ece3.10096

**Published:** 2023-05-18

**Authors:** Olivia M. Williamson, Alexander T. Mustard, Allan J. Bright, Dana E. Williams, Mark C. Ladd, Andrew C. Baker

**Affiliations:** ^1^ Department of Marine Biology and Ecology, Rosenstiel School of Marine, Atmospheric, and Earth Science University of Miami Miami Florida USA; ^2^ Ocean Insight Ltd Peterborough UK; ^3^ Cooperative Institute for Marine and Atmospheric Studies, Rosenstiel School of Marine, Atmospheric, and Earth Science University of Miami Miami Florida USA; ^4^ Southeast Fisheries Science Center, NOAA – National Marine Fisheries Service Miami Florida USA

**Keywords:** coral reef, coral spawning, *Ophioderma rubicundum*, predation, symbiosis, trophic interactions

## Abstract

Many reef invertebrates reproduce through simultaneous broadcast spawning, with an apparent advantage of overwhelming potential predators and maximizing propagule survival. Although reef fish have been observed to consume coral gamete bundles during spawning events, there are few records of such predation by benthic invertebrates. Here, we document several instances of the ruby brittle star, *Ophioderma rubicundum*, capturing and consuming egg‐sperm bundles of the mountainous star coral, *Orbicella faveolata*, and the symmetrical brain coral, *Pseudodiploria strigosa*, during spawning events in the Cayman Islands in 2012 and the Florida Keys in 2022. These observations are widely separated in space and time (>600 km, 10 years), suggesting that this behavior may be prevalent on western Atlantic reefs. Since *O. rubicundum* spawns on the same or subsequent nights as these coral species, we hypothesize that this opportunistic feeding behavior takes advantage of lipid‐rich coral gamete bundles to recover energy reserves expended by the brittle star during gametogenesis. The consumption of coral gametes by adult brittle stars suggests an underexplored trophic link between reef invertebrates and also provides evidence that ophiuroid–coral symbioses may oscillate between commensalism and parasitism depending on the ontogeny and reproductive status of both animals. Our observations provide insights into the nuanced, dynamic associations between coral reef invertebrates and may have implications for coral reproductive success and resilience.

## Graphical abstract

Here, we document several instances of the ruby brittle star, *Ophioderma rubicundum*, capturing and consuming egg‐sperm bundles of two scleractinian coral species during coral spawning events in the Cayman Islands and the Florida Keys. The consumption of coral gametes by adult brittle stars suggests an understudied trophic link between reef invertebrates and also provides evidence that ophiuroid–coral symbioses may shift between commensalism and parasitism depending on the ontogeny and reproductive status of both animals.

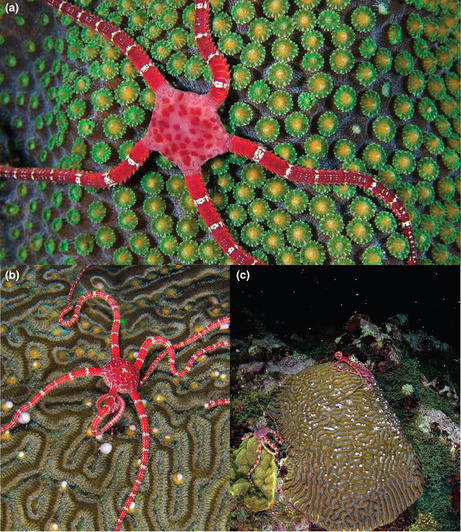

## 1 INTRODUCTION

Most reef‐building scleractinian coral species reproduce through broadcast spawning, whereby gametes are released into the water column for external fertilization (Baird et al., 2009). In many cases, multiple coral species and invertebrate taxa spawn in near synchrony, within minutes or hours of one an other (Babcock et al., 1992, 1986; Bouwmeester et al., 2016; Harrison et al., 1984; Van Veghel, 1993). Mass spawning may have evolved to maximize fertilization success by generating high concentrations of gametes (Levitan et al., 2011; Moláček et al., 2012; Oliver & Babcock, 1992), but also as a strategy to minimize predation losses by saturating predator feeding capacity and reducing the impact on any single spawning individual or species (Alino & Col, 1989; Harrison et al., 1984; Hughes et al., 2000).

On the Great Barrier Reef, planktivorous fish in the families Caesionidae (fusiliers), Chaetodontidae (butterflyfishes), and Pomacentridae (damselfishes) have been found to feed on substantial quantities of coral propagules during mass spawning events (Alino & Col, 1989; Baird et al., 2001; McCormick, 2003; Pratchett et al., 2001; Westneat & Resing, 1988). In the western Atlantic, butterflyfishes have been reported to prey intensely on *Diploria labyrinthiformis* gamete bundles as they are released (Chamberland et al., 2017; Muller & Vermeij, 2011). Although these trophic links between fish and corals are well established (Pratchett et al., 2001), there are remarkably few publications describing consumption of coral spawn by invertebrates or other marine organisms (Schmahl et al., 2008).

Ophiuroids, commonly known as brittle stars, are among the most biodiverse and prolific invertebrates on Caribbean coral reefs (Kissling & Taylor, 1977; Stöhr et al., 2022). Despite their abundance, these cryptic animals are rarely seen, hidden within the reef structure by day and primarily emerging at night to feed (Birkeland, 1988; Fell, 1966; Hendler et al., 1995; Pomory, 2003). The ruby brittle star, *Ophioderma rubicundum*, is widely distributed on shallow reefs throughout the western Atlantic (Clark, 1933; Hotchkiss, 1982; Lewis & Bray, 1983; Pomory, 2003). In some locations such as Carrie Bow Cay, Belize, *O. rubicundum* has been reported among the most numerous ophiuroid species, comprising nearly half of all brittle star specimens collected from fore‐reef environments with high living coral cover (Hendler & Pawson, 2000; Hendler & Peck, 1988). However, this species has been reported at lower abundances (i.e., 0.2 individuals per square meter [Lewis & Bray, 1983]), and is frequently found in reef crest, reef flat, and rubble environments. They have been described as “opportunistic omnivores” that feed on small organisms, including dinoflagellates, diatoms, foraminiferans, hydroids, polychaetes, crustaceans and mollusks (Binyon, 1972; Reese, 1966). In some cases, *O. rubicundum* have been reported to exhibit predatory behavior, seizing prey from the benthos or water column by coiling an arm around it before transporting it to the mouth (Birkeland, 1988; Hendler et al., 1995; Reese, 1966; Reimer & Reimer, 1975). Here, we report observations of *O. rubicundum* capturing and feeding on coral egg‐sperm bundles in two locations in the western Atlantic.

## 2 FIELD OBSERVATIONS

### 2.1 Cayman Islands, September 2012

**FIGURE 1:**
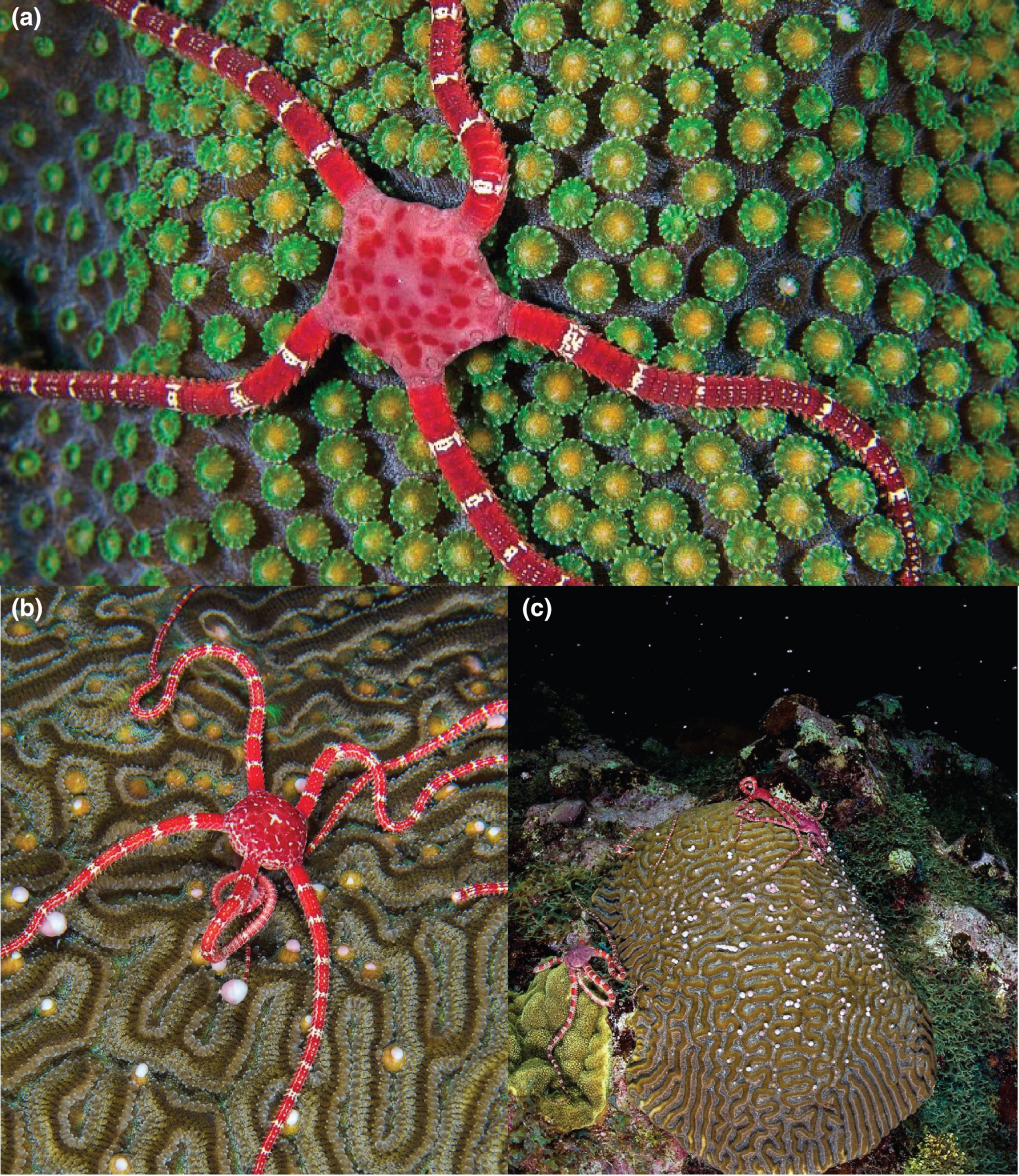
Adult *Ophioderma rubicundum* climbing atop spawning scleractinian corals in Half Moon Bay, East End, Grand Cayman on 6th September 2012. (a) An adult *O. rubicundum* waits on the surface of an *Orbicella faveolata* colony as it stages gamete bundles in the mouths of each polyp, preparing to spawn. (b,c) Four *O. rubicundum* feed on *Pseudodiploria strigosa* gamete bundles as they are released, coiling an arm around the prey before passing it to the mouth.

On September 6, 2012, five nights after the full moon (AFM), divers observed a coral spawning event at Ironshore Gardens in Half Moon Bay, East End, Grand Cayman (19°17′29.1″ N, 81°08′37.1″ W). Throughout the dive, five adult *Ophioderma rubicundum* were photographed climbing atop spawning coral colonies. At 22:13, one *O. rubicundum* was observed to crawl onto the surface of an *Orbicella faveolata* colony as it readied its gamete bundles for release (Figure 1a). From 22:26 to 22:32, four *O. rubicundum* were photographed consuming gamete bundles of a *Pseudodiploria strigosa* colony as it spawned (Figure 1b,c). In both coral species, the brittle stars captured multiple gamete bundles at once with one or two arms, using the arm‐coil behavior described by Reimer and Reimer (1975).

### 2.2 Florida Keys, USA, August 2022

**FIGURE 2:**
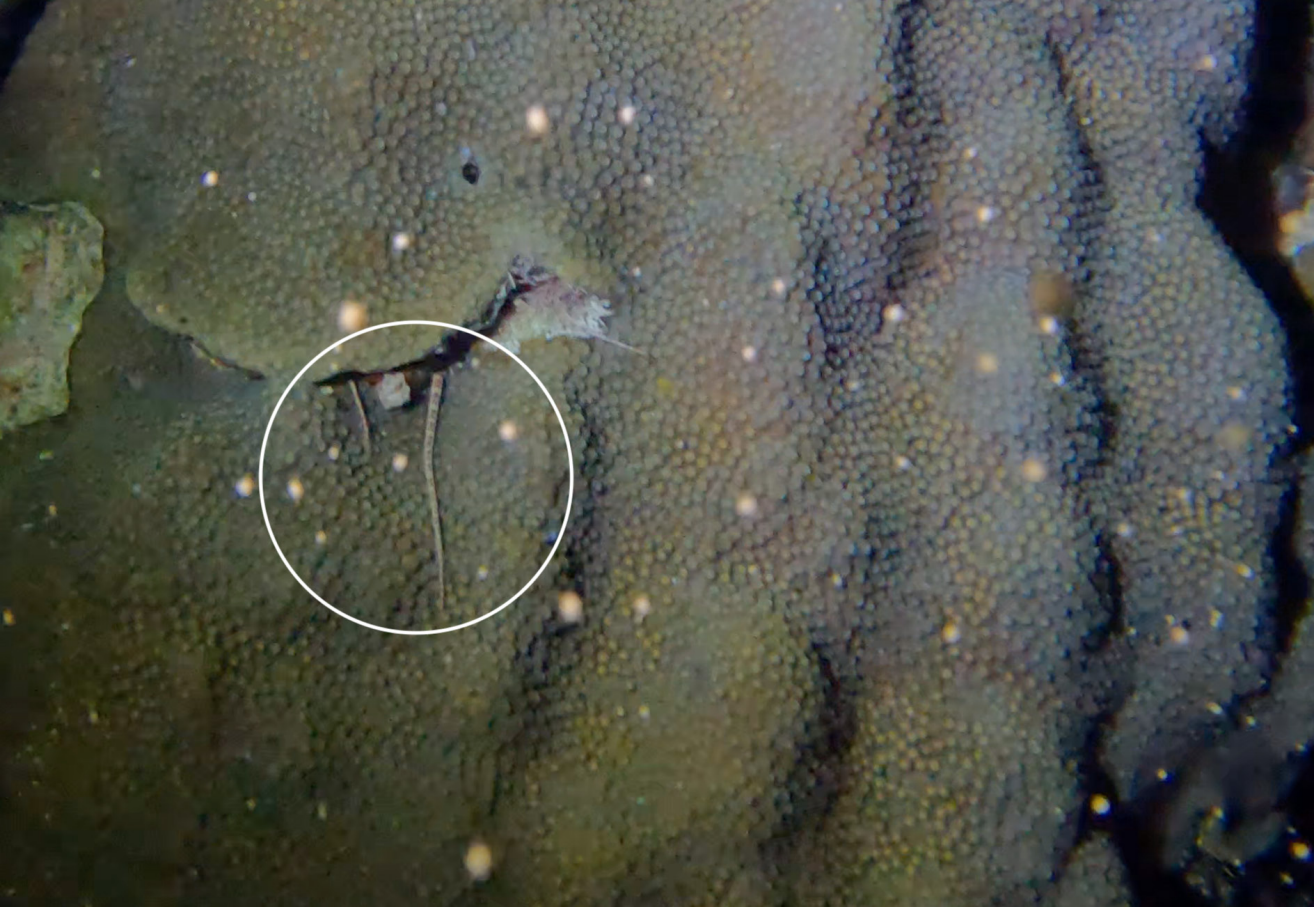
Screenshot from video footage depicting *Ophioderma rubicundum* (circled in white) emerging from its crevice within an *Orbicella faveolata* colony as the coral begins to spawn off Key Largo, FL in August 2022. Arm‐waving behavior from *O. rubicundum* can be seen as parts of the coral has released gamete bundles, while polyps in the area near the brittle star are still “staging” gamete bundles for release.

On August 17, 2022, six nights AFM, divers from the National Oceanic and Atmospheric Administration Southeast Fisheries Science Center (NOAA SEFSC), and the University of Miami Rosenstiel School of Marine, Atmospheric, and Earth Science observed *O. faveolata* spawning at Horseshoe Reef in Key Largo, FL (24°39′40.26″ N, 80°59′39.06″ W). At 00:14 on 18th August, an adult *O. rubicundum* was filmed extending two arms from its shelter within a spawning *O. faveolata* colony, waving them over the coral as the coral began to release gamete bundles (Figure 2). Additional video footage then shows the same brittle star, having emerged completely and perched on the surface of the colony, capturing numerous egg‐sperm bundles (Video 1). The brittle star coiled two of its arms around approximately a dozen gamete bundles each, and was observed to move the bundles toward the mouth.

**VIDEO 1:** An adult *Ophioderma rubicundum* captures egg‐sperm gamete bundles from a spawning *Orbicella faveolata* colony off Key Largo, FL on 17th August 2022. The brittle star has two arms each coiled around approximately one dozen gamete bundles that were just released from the colony.

## 3 DISCUSSION

This report presents rare visual documentation and description of benthic invertebrates consuming gamete bundles during coral spawning events, and represents the first record of this behavior involving multiple coral species and in multiple locations in the western Atlantic.

Despite numerous studies detailing predation by reef fish on coral spawn on the Great Barrier Reef (Alino & Col, 1989; Baird et al., 2001; McCormick, 2003; Pratchett et al., 2001; Westneat & Resing, 1988), there are few accounts of such predation in the western Atlantic (Chamberland et al., 2017; Muller & Vermeij, 2011). Only one publication mentions coral spawn consumption by benthic invertebrates; Schmahl et al. (2008) include one sentence about *Ophioderma rubicundum* collecting coral gamete bundles during a mass spawning event in the Flower Garden Banks (Schmahl et al., 2008), accompanied by a photograph depicting one brittle star atop a spawning *Orbicella franksi* colony (credited to E. L. Hickerson). The addition of our observations from locations as far apart as Key Largo in the Florida Straits and the Cayman Islands in the Caribbean Sea (distances of >600 km), and over decadal timescales (2012 to 2022) suggest that consumption of coral gametes by *O. rubicundum* may be common on Caribbean reefs. In addition, the documented consumption of gamete bundles from multiple species of coral, including *Pseudodiploria strigosa*, *Orbicella faveolata*, and *Orbicella franksi* [Schmahl et al., 2008], indicates that *O. rubicundum* may opportunistically feed on the spawn of any broadcast spawning corals upon or near which they reside.

The specific feeding response we document here—whereby brittle stars coil their arms around gamete bundles—matches how *O. rubicundum* respond to other high‐value food sources. In a laboratory study, *O. rubicundum* presented with pieces of crab, fish meat, and sea urchin viscerae quickly left their hiding places, moved toward the food source, coiled an arm around it, and transported it to the mouth for ingestion (Reimer & Reimer, 1975). The arm‐coiling behavior in our images and footage thus represents a strong feeding response, suggesting that the brittle stars perceive coral gamete bundles as desirable prey items.

This behavior may be related to the reproductive status of each animal, since corals and brittle stars often spawn on the same or subsequent nights. *Orbicella faveolata* and *P. strigosa* typically spawn several hours after sunset, five to nine nights after the full moon (AFM) in August and/or September (Sánchez et al., 1999; Szmant, 1986; Vize et al., 2005; Wyers et al., 1991), while *O. rubicundum* spawns after sunset six to nine nights AFM from August to November (De Graaf et al., 1999; Hagman et al., 1998; Hagman & Vize, 2003; Hendler, 1979; Schmahl et al., 2008). The seasonal production of eggs and sperm is energetically costly for iteroparous invertebrates, requiring considerable investment of resources and space within the body cavity (Greenfield et al., 1958; Giese, 1966; Leuzinger et al., 2003; Stimson, 1987; Ward, 1995). These corals and ophiuroids both release eggs and sperm into the water column for external fertilization (Fell, 1966; Hendler et al., 1995), resulting in planktonic, lecithotrophic larvae rich in polar lipids, wax esters, and triacylglycerols for buoyancy and development (Figueiredo et al., 2012; Giese, 1966; Nevenzel, 1970; Hendler, 1979; Stimson, 1987; Arai et al., 1993; Villinski et al., 2002; Harii et al., 2007; Harii et al., 2010; Padilla‐Gamiño et al., 2013).

Given the nearly concurrent timing of spawning in *O. rubicundum* and various western Atlantic corals, we hypothesize that feeding on coral gamete bundles can supplement the depleted lipid stores of brittle stars and boost metabolic and/or reproductive function (Giese, 1966; Greenfield et al., 1958). On the Great Barrier Reef, planktivorous fishes amass considerable lipid stores as a result of coral gamete consumption (Pratchett et al., 2001). Female *Pomacentrus amboinensis* that feed on large quantities of coral propagules produce larvae with larger yolk sacs and oil globules than those that eat few or none (McCormick, 2003). Presumably, gravid *O. rubicundum* experience similar positive maternal effects from feeding on lipid‐rich coral spawn. Indirect coral gamete consumption may also provide nutrition for reef organisms, evidenced by observations of targeted corallivory on gravid polyps by parrotfish, spider crabs, and butterflyfish (Bright & Miller, 2016; Chamberland et al., 2017; Rotjan & Lewis, 2009). Overall, coral spawning seems to present a convenient and valuable food source to enhance metabolic and/or reproductive output in other reef organisms.

*Ophioderma rubicundum* live inside reef structures, relying on coral colonies as habitat and shelter during daylight hours (Hendler et al., 1995; Pomory, 2003), and perch atop colonies to release their gametes when they spawn (Schmahl et al., 2008). They are typically considered commensals, since no costs or benefits are apparent for their coral hosts. However, our observations warrant a reevaluation of how *O. rubicundum* associate with corals, potentially shifting from commensals to parasites/predators during certain critical times of the year. Similarly dynamic symbioses have been documented among other reef invertebrates, at times impacted by the life stage of both animals. For instance, although cleaning behavior of the obligate sponge‐dwelling brittle star *Ophiothrix lineata* may benefit its host, *Callyspongia vaginalis*, by increasing filtration efficiency (Hendler, 1984), the ophiuroid has also been found to consume the larvae of the sponge, thus exhibiting characteristics of both mutualism and parasitism (Henkel & Pawlik, 2014). In addition, only larger echinoderms take shelter as commensals on or within coral colonies, as larvae and juveniles are vulnerable to tentacle capture or entanglement with coral mucus (Hendler & Littman, 1986; Lewis & Price, 1975; Yamaguchi, 1974). Similar shifts as organisms age may occur in epizoic bryozoans (e.g., *Hippoporidra*) and scleractinians living on shells of hermit crabs. As these epizoites grow, they enlarge the internal habitable volume of the shell, prolonging the use of the shell by the growing crab, effectively shifting the role of the epizoite from commensal to mutualist (Taylor, 2009).

These observations of predation on coral gametes by adult brittle stars highlight the complexity of trophic dynamics during ontogeny and represent an understudied pathway of energy transfer among reef invertebrates. In light of these findings, we suggest that further observations be made during coral spawning events, by divers and/or submersible camera traps, to identify whether additional instances of coral gamete consumption have gone undocumented, especially by other invertebrate species that reproduce around the same time as corals.

Most broadcast spawning corals, including *O. faveolata* and *P. strigosa*, release gametes on just a few nights per year (Szmant, 1986), limiting their prospects for reproduction. Although reef fish can consume large quantities of coral gametes during mass spawning events (Westneat & Resing, 1988), predation occurs in the water column and is likely to be distributed among the gametes of many colonies. In contrast, since brittle stars are confined to the surfaces of coral colonies, their feeding is presumably confined to the gamete bundles of the very coral that it is using for shelter, potentially impacting that individual more directly. Although any loss of gametes, by definition, decreases reproductive fitness, the overall impact of ophiuroid predation remains unknown and likely depends on (1) coral colony size, (2) the number of bundles released during a spawning event, (3) how many brittle stars feed on the surface of the coral, and (4) how many bundles each brittle star consumes. Coral fecundity increases disproportionately with colony size (Hall & Hughes, 1996; Álvarez‐Noriega et al., 2016), likely because smaller colonies invest more energy in somatic growth than reproduction. In large colonies that release thousands of gametes at once, the capture of a few dozen bundles by ophiuroids may be minimally impactful. However, the reproductive success of a smaller colony that releases fewer bundles may diminish considerably if several dozen are eaten upon release, or if multiple brittle stars feed on its surface (as we observed on a *P. strigosa* colony). Consequently, future studies should quantify the number of coral gamete bundles that mature brittle stars consume, particularly as a proportion of total colony output.

The two coral species whose gametes were consumed are particularly vulnerable; *O. faveolata* was listed as “threatened” under the US Endangered Species Act in 2014, and *P. strigosa* was recently reclassified as “critically endangered” by IUCN (Rodríguez‐Martínez et al., 2022), having experienced considerable declines in recent years due to stony coral tissue loss disease (Camacho‐Vite et al., 2022). Coral reproduction is already compromised; as the number of potential parent corals declines and spawning becomes less synchronized (Gardner et al., 2003; Levitan & McGovern, 2005; Shlesinger & Loya, 2019), external pressures such as gamete predation may further decrease fertilization success, reduce recruitment, and inhibit community recovery following disturbance (Hughes et al., 2000; Hughes & Tanner, 2000; Oliver & Babcock, 1992). Consequently, trophic interactions between reef invertebrates may have increasingly important implications for coral reproduction and resilience, warranting further investigation into their nuances.

## AUTHOR CONTRIBUTIONS

**Olivia M. Williamson:** Conceptualization (lead); project administration (lead); supervision (lead); visualization (equal); writing – original draft (lead); writing – review and editing (equal). **Alexander T. Mustard:** Resources (supporting); visualization (equal); writing – review and editing (supporting). **Allan J. Bright:** Project administration (supporting); writing – review and editing (supporting). **Dana E. Williams:** Project administration (supporting); writing – review and editing (supporting). **Mark C. Ladd:** Funding acquisition (equal); project administration (supporting); writing – review and editing (supporting). **Andrew C. Baker:** Conceptualization (supporting); funding acquisition (equal); writing – review and editing (equal).

## Data Availability

Data sharing not applicable to this article as no datasets were generated or analyzed during the current study.

## References

[ece310096-bib-0070] Álvarez‐Noriega, M. , Baird, A. H. , Dornelas, M. , Madin, J. S. , Cumbo, V. R. , & Connolly, S. R. (2016). Fecundity and the demographic strategies of coral morphologies. Ecology, 97(12), 3485–3493. 10.1002/ecy.1588 27912010

[ece310096-bib-0001] Alino, P. M. , & Col, J. C. (1989). Observations of the synchronized mass spawning and post settlement activity of octocorals on the great barrier reef, Australia: Biological aspects. Bulletin of Marine Science, 45(3), 697–707.

[ece310096-bib-0002] Arai, T. , Kato, M. , Heyward, A. , Ikeda, Y. , Iizuka, T. , & Maruyama, T. (1993). Lipid composition of positively buoyant eggs of reef building corals. Coral Reefs, 12, 71–75.

[ece310096-bib-0003] Babcock, R. , Mundy, C. , Keesing, J. , & Oliver, J. (1992). Predictable and unpredictable spawning events: In situ behavioural data from free‐spawning coral reef invertebrates. Invertebrate Reproduction and Development, 22(1–3), 213–227. 10.1080/07924259.1992.9672274

[ece310096-bib-0004] Babcock, R. C. , Bull, G. D. , Harrison, P. L. , Heyward, A. J. , Oliver, J. K. , Wallace, C. C. , & Willis, B. L. (1986). Synchronous spawnings of 105 scleractinian coral species on the great barrier reef. Marine Biology, 90(3), 379–394. 10.1007/BF00428562

[ece310096-bib-0005] Baird, A. H. , Guest, J. R. , & Willis, B. L. (2009). Systematic and biogeographical patterns in the reproductive biology of scleractinian corals. Annual Review of Ecology, Evolution, and Systematics, 40, 551–571. 10.1146/annurev.ecolsys.110308.120220

[ece310096-bib-0006] Baird, A. H. , Pratchett, M. S. , Gibson, D. J. , Koziumi, N. , & Marquis, C. P. (2001). Variable palatability of coral eggs to a planktivorous fish. Marine and Freshwater Research, 52, 865–868.

[ece310096-bib-0007] Binyon, J. (1972). Physiology of echinoderms. Pergamon Press.

[ece310096-bib-0008] Birkeland, C. (1988). The influence of echinoderms on coral‐reef communities . Retrieved from https://www.researchgate.net/publication/284657222. Accessed December 13, 2022.

[ece310096-bib-0009] Bouwmeester, J. , Gatins, R. , Giles, E. C. , Sinclair‐Taylor, T. H. , & Berumen, M. L. (2016). Spawning of coral reef invertebrates and a second spawning season for scleractinian corals in the Central Red Sea. Invertebrate Biology, 135(3), 273–284. 10.1111/IVB.12129

[ece310096-bib-0010] Bright, A. J. , & Miller, M. M. (2016). Foraging by a Caribbean spider crab on the threatened coral, *Acropora palmata*, during coral spawning. Reef Encounter, 31(2), 41–42. 10.53642/OGHN7156

[ece310096-bib-0011] Camacho‐Vite, C. , Estrada‐Saldívar, N. , Pérez‐Cervantes, E. , & Alvarez‐Filip, L. (2022). Differences in the progression rate of SCTLD in *Pseudodiploria strigosa* are related to colony size and morphology. Frontiers in Marine Science, 9, 790818. 10.3389/FMARS.2022.790818

[ece310096-bib-0012] Chamberland, V. F. , Snowden, S. , Marhaver, K. L. , Petersen, D. , & Vermeij, M. J. A. (2017). The reproductive biology and early life ecology of a common Caribbean brain coral, *Diploria labyrinthiformis* (Scleractinia: Faviinae). Coral Reefs, 36(1), 83–94. 10.1007/S00338-016-1504-2/FIGURES/4

[ece310096-bib-0013] Clark, H. L. (1933). A handbook of the Littoral echinoderms of Porto Rico and the other west Indian Islands. New York Academy of Science.

[ece310096-bib-0014] De Graaf, M. , Geertjes, G. J. , & Videler, J. J. (1999). Observations on spawning of scleractinian corals and other invertebrates on the reefs of Bonaire (Netherlands Antilles, Caribbean). Bulletin of Marine Science, 64(1), 189–194.

[ece310096-bib-0015] Fell, H. B. (1966). The ecology of ophiuroids. In R. A. Boolootian (Ed.), Physiology of Echinodermata (pp. 129–143). John Wiley & Sons, Ltd.

[ece310096-bib-0068] Figueiredo, J. , Baird, A. H. , Cohen, M. F. , Flot, J.‐F. , Kamiki, T. , Meziane, T. , Tsuchiya, M. , & Yamasaki, H. (2012). Ontogenetic change in the lipid and fatty acid composition of scleractinian coral larvae. Coral Reefs, 31(2), 613–619. 10.1007/s00338-012-0874-3

[ece310096-bib-0016] Gardner, T. A. , Cote, I. M. , Gill, J. A. , Grant, A. , & Watkinson, A. R. (2003). Long‐term region‐wide declines in Caribbean corals. Science, 301(5635), 958–960. 10.1126/science.1086050 12869698

[ece310096-bib-0017] Giese, A. C. (1966). Lipids in the economy of marine invertebrates. Physiological Reviews, 46(2), 244–298. 10.1152/PHYSREV.1966.46.2.244 5325970

[ece310096-bib-0019] Greenfield, L. , Giese, A. C. , Farmanfarmaian, A. , & Boolootian, R. A. (1958). Cyclic biochemical changes in several echinoderms. Journal of Experimental Zoology, 139(3), 507–524. 10.1002/JEZ.1401390308

[ece310096-bib-0020] Hagman, D. K. , Gittings, S. R. , Deslarzes, K. J. P. , Hagman, D. K. , & Gittings, S. R. (1998). Timing, species participation, and environmental factors influencing annual mass spawning at the flower garden banks (Northwest Gulf of Mexico). Gulf of Mexico Science, 16(2), 170–179. 10.18785/goms.1602.06

[ece310096-bib-0021] Hagman, D. K. , & Vize, P. D. (2003). Mass spawning by two brittle star species, *Ophioderma rubicundum* and *O. Squamosissimum* (Echinodermata: Ophiuroidea), at the flower garden banks, Gulf of Mexico. Bulletin of Marine Science, 72(3), 871–876.

[ece310096-bib-0069] Hall, V. R. , & Hughes, T. P. (1996). Reproductive strategies of modular organisms: Comparative studies of reef‐building corals. Ecology, 77(3), 950–963. 10.2307/2265514

[ece310096-bib-0022] Harii, S. , Nadaoka, K. , Yamamoto, M. , & Iwao, K. (2007). Temporal changes in settlement, lipid content and lipid composition of larvae of the spawning hermatypic coral *Acropora tenuis* . Marine Ecology Progress Series, 346, 89–96. 10.3354/MEPS07114

[ece310096-bib-0023] Harii, S. , Yamamoto, M. , & Hoegh‐Guldberg, O. (2010). The relative contribution of dinoflagellate photosynthesis and stored lipids to the survivorship of symbiotic larvae of the reef‐building corals. Marine Biology, 157(6), 1215–1224. 10.1007/S00227-010-1401-0/TABLES/3

[ece310096-bib-0024] Harrison, P. L. , Babcock, R. C. , Bull, G. D. , Oliver, J. K. , Wallace, C. C. , & Willis, B. L. (1984). Mass spawning in tropical reef corals. Science, 223(4641), 1186–1189. 17742935 10.1126/science.223.4641.1186

[ece310096-bib-0025] Hendler, G. (1979). Reproductive periodicity of ophiuroids (Echinodermata: Ophiuroidea) on the Atlantic and Pacific coasts of Panamá. In S. E. Stancyk (Ed.), Reproductive ecology of marine invertebrates (pp. 145–156). University of South Carolina Press.

[ece310096-bib-0026] Hendler, G. (1984). The association of *Ophiothrix lineata* and *Callyspongia vaginali*s: A brittlestar‐sponge cleaning symbiosis? Marine Ecology, 5(1), 9–27. 10.1111/J.1439-0485.1984.TB00304.X

[ece310096-bib-0027] Hendler, G. , & Littman, B. S. (1986). The ploys of sex: Relationships among the mode of reproduction, body size and habitats of coral‐reef brittlestars. Coral Reefs, 5, 31–42.

[ece310096-bib-0028] Hendler, G. , Miller, J. E. , Pawson, D. L. , & Kier, P. M. (1995). Sea stars, sea urchins, and allies: Echinoderms of Florida and the Caribbean. Smithsonian Institution Press.

[ece310096-bib-0029] Hendler, G. , & Pawson, D. L. (2000). Echinoderms of the rhomboidal cays, Belize: Biodiversity, distribution, and ecology. Atoll Research Bulletin, 479, 275–299 Retrieved from https://repository.si.edu/bitstream/handle/10088/33941/Atoll_479_Echinoderms.pdf. Accessed December 13, 2022.

[ece310096-bib-0030] Hendler, G. , & Peck, R. W. (1988). Ophiuroids off the deep end: Fauna of the Belizean fore‐reef slope. In R. D. Burke , P. V. Mladenov , P. Lambert , & R. L. Parsley (Eds.), Echinoderm biology: Proceedings of the sixth international echinoderm conference, Victoria, 23–28 august 1987 (pp. 411–419). A. A. Balkema.

[ece310096-bib-0031] Henkel, T. P. , & Pawlik, J. R. (2014). Cleaning mutualist or parasite? Classifying the association between the brittlestar *Ophiothrix lineata* and the Caribbean reef sponge *Callyspongia vaginalis* . Journal of Experimental Marine Biology and Ecology, 454, 42–48. 10.1016/j.jembe.2014.02.005

[ece310096-bib-0032] Hotchkiss, F. H. C. (1982). Ophiuroidea (Echinodermata) from Carrie bow cay, Belize. In K. Rützler & I. G. Macintyre (Eds.), The Atlantic barrier reef ecosystem at Carrie bow cay, Belize, 1: Structure and communities (pp. 387–412). Smithsonian Institution Press.

[ece310096-bib-0033] Hughes, T. P. , Baird, A. H. , Dinsdale, E. A. , Moltschaniwskyj, N. A. , Pratchett, M. S. , Tanner, J. E. , & Willis, B. L. (2000). Supply‐side ecology works both ways: The link between benthic adults, fecundity, and larval recruits. Ecology, 81(8), 2241–2249. 10.2307/177111

[ece310096-bib-0034] Hughes, T. P. , & Tanner, J. E. (2000). Recruitment failure, life histories, and long‐term declines of Caribbean corals. Ecology, 8(8), 2250–2263. 10.2307/177112

[ece310096-bib-0035] Kissling, D. L. , & Taylor, G. T. (1977). Habitat factors for reef dwelling ophiuroids in the Florida keys. *3rd international coral reef symposium*, 225–231.

[ece310096-bib-0037] Leuzinger, S. , Anthony, K. R. N. , & Willis, B. L. (2003). Reproductive energy investment in corals: Scaling with module size. Oecologia, 136(4), 524–531. 10.1007/S00442-003-1305-5/TABLES/5 12802676

[ece310096-bib-0038] Levitan, D. R. , & McGovern, T. M. (2005). The allee effect in the sea. In E. A. Norse & L. B. Crowder (Eds.), Marine conservation biology (pp. 47–57). Island Press.

[ece310096-bib-0039] Lewis, J. B. , & Bray, R. D. (1983). Community structure of ophiuroids (Echinodermata) from three different habitats on a coral reef in Barbados, West Indies. Marine Biology, 73, 171–176. 10.1007/BF00406885

[ece310096-bib-0040] Lewis, J. B. , & Price, W. S. (1975). Feeding mechanisms and feeding strategies of Atlantic reef corals. Journal of Zoology, 176(4), 527–544. 10.1111/J.1469-7998.1975.TB03219.X

[ece310096-bib-0066] Levitan, D. R. , Fogarty, N. D. , Jara, J. , Lotterhos, K. E. , & Knowlton, N. (2011). Genetic, spatial, and temporal components of precise spawning synchrony in reef building corals of the *Montastraea annularis* complex. Evolution, 65(5), 1254–1270. 10.1111/j.1558-5646.2011.01235.x 21521188

[ece310096-bib-0041] McCormick, M. I. (2003). Consumption of coral propagules after mass spawning enhances larval quality of damselfish through maternal effects. Oecologia, 136(1), 37–45. 10.1007/S00442-003-1247-Y 12707838

[ece310096-bib-0042] Moláček, J. , Denny, M. , & Bush, J. W. M. (2012). The fine art of surfacing: Its efficacy in broadcast spawning. Journal of Theoretical Biology, 294, 40–47. 10.1016/J.JTBI.2011.10.013 22019506

[ece310096-bib-0043] Muller, E. , & Vermeij, M. J. A. (2011). Day time spawning of a Caribbean coral. Coral Reefs, 30(4), 1147. 10.1007/S00338-011-0814-7/FIGURES/1

[ece310096-bib-0044] Nevenzel, J. C. (1970). Occurrence, function and biosynthesis of wax esters in marine organisms. Lipids, 5(3), 308–319. 10.1007/BF02531462 4392482

[ece310096-bib-0045] Oliver, J. , & Babcock, R. (1992). Aspects of the fertilization ecology of broadcast spawning corals: Sperm dilution effects and in situ measurements of fertilization. Biological Bulletin, 183(3), 409–417. 10.2307/1542017 29300507

[ece310096-bib-0046] Padilla‐Gamiño, J. L. , Bidigare, R. R. , Barshis, D. J. , Alamaru, A. , Hédouin, L. , Hernández‐Pech, X. , Kandel, F. , Soon, S. L. , Roth, M. S. , Rodrigues, L. J. , Grottoli, A. G. , Portocarrero, C. , Wagenhauser, S. A. , Buttler, F. , & Gates, R. D. (2013). Are all eggs created equal? A case study from the Hawaiian reef‐building coral *Montipora capitata* . Coral Reefs, 32(1), 137–152. 10.1007/S00338-012-0957-1/TABLES/4

[ece310096-bib-0047] Pomory, C. M. (2003). A guide to the shallow‐water Echinodermata of the Texas coast. Contributions in Marine Science, 36, 1–188 Retrieved from https://repositories.lib.utexas.edu/bitstream/handle/2152/18141/Contributions_Volume36.pdf?sequence=2&isAllowed=y. Accessed December 13, 2022.

[ece310096-bib-0048] Pratchett, M. S. , Gust, N. , Goby, G. , & Klanten, S. O. (2001). Consumption of coral propagules represents a significant trophic link between corals and reef fish. Coral Reefs, 20(1), 13–17. 10.1007/S003380000113

[ece310096-bib-0049] Reese, E. S. (1966). The complex behavior of echinoderms. In R. A. Boolootian (Ed.), Physiology of Echinodermata (pp. 157–218). John Wiley & Sons, Ltd.

[ece310096-bib-0050] Reimer, R. D. , & Reimer, A. A. (1975). Chemical control of feeding in four species of tropical ophiuroids of the genus *Ophioderma* . Comparative Biochemistry and Physiology Part A: Physiology, 51(4), 915–927. 10.1016/0300-9629(75)90075-4 237718

[ece310096-bib-0051] Rodríguez‐Martínez, R. , Banaszak, A. , Crabbe, J. , & Vermeij, M. (2022). Pseudodiploria strigosa . The IUCN Red List of Threatened Species 2022: e.T133155A165745174. Accessed on 15 December 2022.

[ece310096-bib-0052] Rotjan, R. D. , & Lewis, S. M. (2009). Predators selectively graze reproductive structures in a clonal marine organism. Marine Biology, 156, 569–577. 10.1007/s00227-008-1108-7

[ece310096-bib-0053] Sánchez, J. A. , Alvarado, E. M. , Gil, M. F. , Charry, H. , Arenas, O. L. , Chasqui, L. H. , & García, R. P. (1999). Synchronous mass spawning of *Montastraea annularis* (Ellis & Solander) and *Montastraea faveolata* (Ellis & Solander) (Faviidae: Scleractinia) at Rosario Islands, Caribbean coast of Colombia. Bulletin of Marine Science, 65(3), 873–879.

[ece310096-bib-0054] Schmahl, G. P. , Hickerson, E. L. , & Precht, W. F. (2008). Biology and ecology of coral reefs and coral communities in the flower garden banks region, northwestern Gulf of Mexico. In B. M. Riegl & R. E. Dodge (Eds.), Coral reefs of the USA. Coral reefs of the world (Vol. 1). Springer. 10.1007/978-1-4020-6847-8_6

[ece310096-bib-0055] Shlesinger, T. , & Loya, Y. (2019). Breakdown in spawning synchrony: A silent threat to coral persistence. Science, 365(6457), 1002–1007. 10.1126/SCIENCE.AAX0110 31488683

[ece310096-bib-0056] Stimson, J. S. (1987). Location, quantity and rate of change in quantity of lipids in tissue of Hawaiian hermatypic corals. Bulletin of Marine Science, 41(3), 889–904.

[ece310096-bib-0057] Stöhr, S. , O'Hara, T. , & Thuy, B. (Eds.). (2022). World Ophiuroidea Database. Accessed at https://www.marinespecies.org/ophiuroidea on 2022‐09‐01 10.14284/358

[ece310096-bib-0058] Szmant, A. M. (1986). Reproductive ecology of Caribbean reef corals. Coral Reefs, 5, 43–54.

[ece310096-bib-0059] Taylor, P. D. (2009). Evolutionary palaeoecology of symbioses between bryozoans and hermit crabs. Historical Biology, 9(3), 157–205. 10.1080/10292389409380497

[ece310096-bib-0060] Van Veghel, M. L. J. (1993). Multiple species spawning on Curaçao reefs. Bulletin of Marine Science, 52(3), 1017–1021.

[ece310096-bib-0061] Villinski, J. T. , Villinski, J. C. , Byrne, M. , & Raff, R. A. (2002). Convergent maternal provisioning and life‐history evolution in echinoderms. Evolution, 56(9), 1764–1775. 10.1111/J.0014-3820.2002.TB00190.X 12389721

[ece310096-bib-0062] Vize, P. D. , Embesi, J. A. , Nickell, M. , Brown, D. P. , & Hagman, D. K. (2005). Tight temporal consistency of coral mass spawning at the flower garden banks, Gulf of Mexico, from 1997‐2003. Gulf of Mexico Science, 23(1), 8. 10.18785/goms.2301.08

[ece310096-bib-0067] Ward, S. (1995). Two patterns of energy allocation for growth, reproduction and lipid storage in the scleractinian coral *Pocillopora damicornis* . Coral Reefs, 14, 87–90.

[ece310096-bib-0063] Westneat, M. W. , & Resing, J. A. M. (1988). Predation on coral spawn by planktivorous fish. Coral Reefs, 7(2), 89–92. 10.1007/BF00301646

[ece310096-bib-0064] Wyers, S. C. , Barnes, H. S. , & Smith, S. R. (1991). Spawning of hermatypic corals in Bermuda: A pilot study. In R. B. Williams , P. F. S. Cornelius , R. G. Hughes , & E. A. Robson (Eds.), Coelenterate biology: Recent research on cnidaria and Ctenophora (pp. 109–116). Springer Dordrecht. 10.1007/978-94-011-3240-4_15

[ece310096-bib-0065] Yamaguchi, M. (1974). Growth of juvenile *Acanthaster planci* (L.) in the laboratory. Pacific Science, 28(2), 123–138.

